# Development of a new model system to dissect isoform specific Akt signalling in adipocytes

**DOI:** 10.1042/BJ20150191

**Published:** 2015-06-15

**Authors:** Esi Kajno, Timothy E. McGraw, Eva Gonzalez

**Affiliations:** * Biochemistry Department, Weill Cornell Medical College, 1300 York Avenue, Room W-320, New York, NY 10065, U.S.A.

**Keywords:** adipogenesis, adipocyte, Akt isoform signalling, insulin action, glucose transporter 4 (GLUT4), MK-2206

## Abstract

Protein kinase B (Akt) kinases are critical signal transducers mediating insulin action. Genetic studies revealed that Akt1 and Akt2 signalling differentially contribute to sustain lipid and glucose homoeostasis; however Akt isoform-specific effectors remain elusive due to the lack of a suitable model system to mechanistically interrogate Akt isoform-specific signalling. To overcome those technical limitations we developed a novel model system that provides acute and specific control of signalling by Akt isoforms. We generated mutants of Akt1 and Akt2 resistant to the allosteric Akt inhibitor MK-2206. We then developed adipocyte cell lines, in which endogenous Akt1 or Akt2 has been replaced by their corresponding drug-resistant Akt mutant. Treatment of those cells with MK-2206 allowed for acute and specific control of either Akt1 or Akt2 function. Our data showed that Akt1^W80A^ and Akt2^W80A^ mutants are resistant to MK-2206, dynamically regulated by insulin and able to signal to Akt downstream effectors. Analyses of insulin action in this cellular system showed that Akt1 and Akt2 are both able to mediate insulin regulation of the transcription factor forkhead box O1 (FoxO1) and the glucose transporter 4 (GLUT4), revealing a redundant role for these Akt kinases in the control of glucose transport into fat cells. In contrast, Akt1 signalling is uniquely required for adipogenesis, by controlling the mitotic clonal expansion (MCE) of pre-adipocytes that precedes white adipose cell differentiation. Our data provide new insights into the role of Akt kinases in glucose transport and adipogenesis and support our model system as a valuable tool for the biochemical characterization of signalling by specific Akt isoforms.

## Abstract (precis)

Our study describes the development and validation of a new model system that allows for acute control of signalling by specific Akt isoforms. This model system revealed new insights into the role of Akt kinases in glucose transport and adipogenesis.

## INTRODUCTION

Akt kinases are a critical signalling node controlling glucose and lipid metabolism in response to insulin [1–3]. This family of kinases is comprised of three highly conserved homologues Akt1, Akt2 and Akt3 [also referred to as protein kinase B (PKB)α, PKBβ and PKBγ respectively; 4]. All three kinases share a common activation mechanism in response to insulin, where they are recruited to the cell surface by receptor-activated phosphatidylinositol (PI)-3 kinase (PI3-kinase) and phosphorylated by the PDK1 (phosphoinositide-dependent kinase-1) and mammalian target of rapamycin (mTOR)C2 kinases [5,6]. Despite their conserved regulatory mechanisms and high sequence homology, Akt isoforms are known to differently contribute to metabolic regulation [4,7].

Although Akt1 and Akt2 are ubiquitously expressed kinases, the Akt2 isoform is more abundant in metabolic tissues. Inactivating mutations in Akt2 correlate with insulin resistance in humans [8] and genetic deletion of Akt2 in mice causes an insulin resistance-like phenotype [9,10]. *In vitro* studies showed that Akt2 signalling regulates glucose homoeostasis by several mechanisms. Akt2 mediates glucose transport into fat and muscle cells by inhibiting the Rab GAP (GTP-hydrolysis activating protein) AS160 (Akt substrate of 160 kDa), which facilitates the plasma membrane translocation of GLUT4 (glucose transporter 4) glucose transporters [9,11–16]. Akt2 signalling also controls glucose levels by blunting hepatic gluconeogenesis through inactivation of the transcription factor FoxO1 (forkhead box O1) [9,17]. Unlike Akt2, genetic inactivation of Akt1 in mice does not result in metabolic defects [18,19]. However, haplodeficiency of Akt1 in Akt2-null mice exacerbates their insulin resistant phenotype to cause overt type 2 diabetes [20], thereby suggesting that Akt1 might also contribute to sustain glucose homoeostasis in mice. Although genetic models have been instrumental in defining Akt kinases’ *in vivo* functions, differences in their expression levels, redundancies between the Akt isoforms in metabolic tissues and potential compensatory mechanisms due to long-term deletion of a specific Akt kinase limit the identification of the molecular mechanisms and effectors by which Akt isoform-specific signalling controls lipid and glucose metabolism. A better molecular understanding of Akt isoform effectors mediating insulin metabolic control holds the promise for the development of new and specific pharmacological interventions to improve insulin action in conditions characterized by defective Akt signalling, like insulin resistance and type 2 diabetes.

To date the discovery of the effector molecules that mediate metabolic functions of specific Akt isoforms has been hampered by our inability to acutely, specifically and robustly inhibit the activity of individual Akt isoforms in one cell type at a time. Chemical genetics, which combines pharmacological inhibitors with engineered drug-resistant as well as sensitive molecules, can provide a temporal and reversible control of signalling intermediates in a cellular context. It thereby presents a powerful approach to investigate the biochemical mechanisms of cell signalling processes [21]. Although Akt inhibitors have long been characterized by a poor specificity, a new generation of potent and specific Akt allosteric inhibitors with partial Akt isoform selectivity has previously been described [22,23]. Combination of one of those allosteric inhibitors, the quinoxaline Akt 1/2 inhibitor (Akti1/2), with an Akt1 mutant resistant to this drug suggested a role for Akt1 in insulin-regulated glucose and amino acid uptake [24]. This finding supports the applicability of chemical genetics for the study of Akt function. However, the poor pharmacokinetics and unspecific effects of the Akti1/2 inhibitor on glucose transport and platelet function limits its use as a research and therapeutic agent [25,26]. Those limitations have been largely overcome by the new MK-2206 compound, which displays enhanced affinity for Akt1 and Akt2, decreased off target effects, improved solubility and superior pharmacokinetics [27–30], thus providing a valuable tool for the characterization of Akt kinase function.

In the present study, we used the MK-2206 inhibitor in combination with Akt1 and Akt2 mutants that resist this drug to generate an adipocyte model system in which signalling by specific Akt isoforms can be acutely and specifically regulated. Analyses of isoform specific Akt functions in our novel model system showed a redundancy between Akt1 and Akt2 signalling in the regulation of GLUT4 trafficking in fat cells and validated a specific requirement for Akt1 signalling in adipocyte differentiation.

## EXPERIMENTAL

### Antibodies, drugs, cDNA constructs

Antibodies against phospho-Akt (Ser^473^ and Thr ^308^), phospho-FoxO1 (Ser^256^), phospho-AS160 (Thr^642^), Akt1, Akt2, phospho-MAPK (mitogen-activated protein kinase) p42/44, MAPK 42/44, perilipin1 and FoxO1 were obtained from Cell Signaling Technologies. Anti-Flag epitope antibody was purchased from Sigma–Aldrich. Anti-AS160, anti-IRS1 (insulin receptor substrate 1) and anti-phosphotyrosine antibodies were from Upstate Biotech. Mouse anti-HA epitope monoclonal antibody was from Covance. Fluorescent secondary antibodies were purchased from Jackson Immunolabs and Invitrogen. Akt inhibitor MK-2206 was purchased from SelleckChem. FoxO1–GFP construct was kindly provided by Dr Domenico Accili (Columbia University, New York). The cDNA constructs encoding Flag–Akt1 and Flag–Akt2 were previously described [14]. Flag-tagged Akt mutants Akt1^W80A^ and Akt2^W80A^ were generated by site-directed mutagenesis using QuickChange II (Agilent Technology). The following primer pairs were used: Akt1^W80A^ forward: 5′-CATCCGCTGCCTGCAGGCGA-CCACAGTCATTGAGCG-3′, reverse: 5′-CGCTCAATGACT-GTGGTCGCCTGCAGGCAGCGGATG-3′; Akt2^W80A^ forward 5′-CATACGCTGCCTGCAGGCGACCACAGTCATCGAGAGG-3′, reverse: 5′-CCTCTCGATGACTGTGGTCGCCTGCAGG-CAGCGTATG-3′. Akt1 and Akt2 shRNA were previously described [31]. Akt1 and Akt2 shRNA-resistant variants of wild-type (WT) and W80A Akt mutants were generated by introducing wobbled mutations into the cDNA of Flag-tagged Akt constructs by site-directed mutagenesis using QuickChange II. The following primer pairs were used: shRNA-resistant Akt1 forward: 5′-CGCTTACTGAGAACCGCGTGCTTCAGAACTCTAGG-3′, reverse: 5′-CCTAGAGTTCTGAAGCACGCGGTTCTCAGT-AAGCG-3′; shRNA-resistant Akt2 forward: 5′-CGGGCCAA-AGTGACCATGAACGATTTCGACTATCTCAAACTCC-3′, reverse: 5′-GGAGTTTGAGATAGTCGAAATCGTTCATGGT-CACTTTGGCCCG-3′. cDNAs encoding Flag-tagged Akt1^WT^ and Akt1^W80A^ resistant to Akt1 shRNA and Flag-tagged Akt2^WT^ and Akt2^W80A^ resistant to Akt2 shRNA were subcloned into the lentiviral vector pLVX-IRES-tdTomato (Clonthec).

### Cell line generation, cell culture, adipocyte differentiation and electroporation

3T3-L1 fibroblasts were cultured, differentiated into adipocytes and electroporated as previously described [32,33]. Experiments were performed on day 5 after differentiation unless noted otherwise. 3T3-L1 cells stably expressing Akt1 shRNA (Akt1^KD^) and Akt2 shRNA (Akt2^KD^) were generated by retroviral infection as previously described [31]. To generate 3T3-L1 stable cell lines expressing WT or drug resistant Akt mutants, Akt1^KD^ cells were infected with lentiviral particles encoding shRNA-resistant Flag-tagged Akt1^WT^ or Akt1^W80A^; Akt2^KD^ cells were infected with lentiviral particles encoding shRNA-resistant Flag-tagged Akt2^WT^ or Akt2^W80A^ using the Lenti-X lentiviral expression system from Clonthec. Infected cells were sorted by flow cytometry based on expression of the fluorescence marker tdTomato. Expression of transgenic Akt constructs was confirmed by indirect immunofluorescence microscopy and Western blot analysis with an anti-Flag epitope antibody. To isolate cell populations with different expression levels of Flag–Akt2^W80A^, the Akt2^W80A^ cell population was sorted by flow cytometry. tdTomato positive cells were gated to isolate cell populations with low (bottom 7%): Akt2^W80A^_Low_; medium: Akt2^W80A^_Medium_; and high (top 5%): Akt2^W80A^_High_ expression levels of the tdTomato fluorescence marker. The expression levels of Akt2^W80A^ in those cell populations were assessed by Western blot analysis with an anti-Akt2 antibody.

### Immunoblot analyses and immunoprecipitation

3T3-L1 adipocytes were starved in serum-free DMEM (Dulbecco's modified Eagle's medium) with 20 mM of sodium bicarbonate, 20 mM HEPES (pH 7.2) at 37°C in 5% CO_2_/air for 3–8 h prior to all experiments. After insulin treatments, 3T3-L1 adipocytes were washed with 150 mM NaCl, 20 mM HEPES, 1 mM CaCl2, 5 mM KCl, 1mM MgCl_2_ (pH 7.2) and lysed in lysis buffer (Cell Signaling Technology). Western blot analyses were performed using standard protocols and densitometric analyses of immunoblots were performed using MetaMorph software. For immunoprecipitation studies protein lysates were incubated with specific antibodies for 1 h followed by incubation with Sepharose-coupled protein A/G beads (Santa Cruz Biotechnology) overnight. Beads were washed three times with lysis buffer and immunoprecipitates were resolved by SDS/PAGE and blotted as described above.

### GLUT4 translocation

Haemagglutinin (HA)–GLUT4–GFP translocation assay has been described in detail [31,32].

### FoxO1 subcellular localization

Subcellular localization of FoxO1 was determined using FoxO1–GFP reporter and fluorescence microscopy [14]. Cells were scored for nuclear or cytosolic FoxO1–GFP localization and results are expressed as the percentage of cells with cytoplasmic FoxO1.

### Adipogenesis studies

To assess the role of Akt signalling in adipocyte differentiation 3T3-L1 pre-adipocytes were grown to confluence, starved for 2 days and differentiated by addition of differentiation induction medium (DMEM–10% FBS, 170 nM insulin, 0.5 mM IBMX (3-isobutyl-1-methylxanthine), 0.25 μM dexamethasone, 1 μM rosiglitazone) in the presence or absence of 2 μM MK-2206. MK-2206 treated cells were supplemented with 2 μM MK-2206 every 24 h. Three days later, culture medium was changed to post-induction medium (DMEM, 10% FBS, 170 nM insulin) with or without 2 μM MK-2206. Adipocytes were subsequently fed every 2 days with DMEM–10% FBS until day 8–10 post-induction when lipid accumulation was assessed and mRNA was collected.

### RT-qPCR analyses

mRNA from adipocytes was isolated using the RNeasy kit from Quiagen and quantified using a Nanodrop spectrophotometer. cDNA was synthesized from 1 μg of total RNA using qScript (Quanta Bioscience). Real-time (RT)-qPCR (quantitative PCR) was performed on a C1000 Thermal Cycler (Bio-Rad) and quantified using Bio-Rad CFX Manager software. The following primer pair sequences were used: *Rplp0* (ribosomal protein, large P0) forward: 5′-GTGCCATCGCCCCGTGTG-3′, reverse 5′-TGGATGATCAGCCCGAAGGAGA-3′; *Prlpn1* (perilipin1) forward 5′-GAGAGGAGACAGACGACGAG-3′, reverse 5′-TCCCTTTGGTAGAGGAGACA-3′; *Pparγ* (peroxisome proliferator-activated receptor gamma) forward 5′-GGAAAGACAACGGACAAATC-3′, reverse 5′-TGGACACCATACTTGAGCAG-3′; *Fabp4* (fatty acid biding protein 4) forward 5′-CACCGAGATTTCCTTCAAAC-3′ reverse 5′-GTCACGCCTTTCATAACACA-3′; *Glut4* forward 5′-CATGA-GCTATGTCTCCATCG-3′, reverse 5′-CTCTGGTTTCAGGCA-CTTTT-3′; *adiponectin* forward 5′-TGTTCCTCTTAAT-CCTGCCCA-3′, reverse 5′-CCAACCTGCACAAGTTCCCTT-3′; *adipsin* forward 5′-CATGCTCGGCCCTACATGG-3′, reverse 5′-CACAGAGTCGTCATCCGTCAC-3′; *cEbpα* (CCAAT/enhancer binding protein alpha) forward 5′-CAAGA-ACAGCAACGAGTACCG-3′, reverse 5′-GTCACTGGTCAA-CTCCAGCAC-3′; *Lpl* (lipoprotein lipase) forward 5′-GGGAGTTTGGCTCCAGAGTTT-3′, reverse 5′-TGTGTCTTCAGGGGTCCTTAG-3′; *Fas* (fatty acid synthase) forward 5′-GCTGGCATTCGTGATGGAGTCGT-3′, reverse 5′-AGGCCACCAGTGATGATGTAACTCT-3′. Primer pair specificity was confirmed by the absence of product in samples prepared without reverse transcriptase; and product sizes confirmed by gel electrophoresis. For each target gene data were normalized relative to large ribosomal protein (*Rplp0*). For each target gene results are expressed as the log_2_-fold change in gene expression in MK-2206-treated compared with -untreated conditions.

### Neutral lipid accumulation measurements

Neutral lipid content in adipocytes was detected using Oil-Red-O and LipidTox staining (Invitrogen). For Oil-Red-O staining cells were fixed in 3.7% formaldehyde for 1 h at room temperature, washed twice with PBS and twice with water, followed by incubation with Oil-Red-O stain solution (60:40; 0.5% Oil-Red-O in isopropanol–water) for 1 h at room temperature. LipidTox staining of neutral lipids was performed and quantified as described in [34].

### EdU (5-ethynyl-2′-deoxyuridine) incorporation analyses

Pre-adipocytes were seeded on coverslip bottom dishes and allowed to reach confluence. Cells were starved for 2 days, followed by induction of differentiation using induction medium with or without MK2206 (2 μM). Sixteen hours after differentiation, cells were pulsed with EdU (10 mM) for 2 h and EdU incorporation was visualized using CLICK-IT EdU Alexa-488 Imaging Kit (Invitrogen). Nuclei were counterstained with Hoechst 3342 and EdU incorporation was measured by fluorescence microscopy. Images were analysed using the MetaMorph cell scoring software application to determine the percentage of EdU positive cells.

### Fluorescence microscopy acquisition and quantification

Fluorescence microscopy was performed using a DMIRB inverted microscope (Leica Microsystems), with a cooled charge-coupled device camera (Princeton Instruments). Images were collected with a 20× objective. To perform ‘prismless’ TIRF microscopy a 60×1.45 numerical aperture oil-immersion objective (Olympus America) was used. The evanescent field decay length was 100–250 nm. MetaMorph software (Universal Imaging) was used for image processing and quantification as previously described [35].

### Statistical analysis

Statistical significance was determined using Student's *t* test and ANOVA as noted.

## RESULTS

### Development of a cellular model system to study Akt isoform function

To investigate the cellular and molecular mechanisms by which Akt kinases relay insulin signals in adipocytes, we developed a novel assay system that enables us to acutely, specifically and reversibly control the activity of individual Akt isoforms. Akt1 and Akt2 kinases are effectively inhibited by the allosteric inhibitors Akti1/2 and MK-2206 [22,23,28]. Structural analyses of Akt1 bound to Akti1/2 revealed a tight interaction between Trp^80^ of Akt1 and the inhibitor [36] and mutation of Trp^80^ to alanine renders Akt1 insensitive to Akti1/2 [24]. Since the Akti1/2 drug inhibits both Akt1 and Akt2 and Trp^80^ is conserved among those kinases, we hypothesized that the Akt2^W80A^ mutant may also resist this inhibitor. To test our hypothesis we developed Flag-tagged Akt1^W80A^ or Akt2^W80A^ mutants and we retrovirally infected adipocytes with each of those constructs or a WT Akt2 construct. We then treated those cells with insulin in the presence or absence of the Akt inhibitors Akti1/2 or MK-2206. Our Western blot analyses of immunoprecipitated Flag-tagged Akt kinases showed that Akt1^W80A^ and Akt2^W80A^ mutants were phosphorylated in response to insulin similar to Akt2^WT^ (Supplementary Figure S1). Treatment with Akti1/2 or MK-2206 drugs inhibited insulin-induced phosphorylation of Akt2^WT^ at Thr^309^ and Ser^474^, whereas phosphorylation of Akt1^W80A^ and Akt2^W80A^ at both regulatory sites remained intact (Supplementary Figure S1). These results suggest that Akt1^W80A^ and Akt2^W80A^ are resistant to both Akti1/2 and MK-2206 drugs. Due to the enhanced specificity, potency and pharmacokinetics of MK-2206 we focused our subsequent studies on this inhibitor.

**Figure 1:**
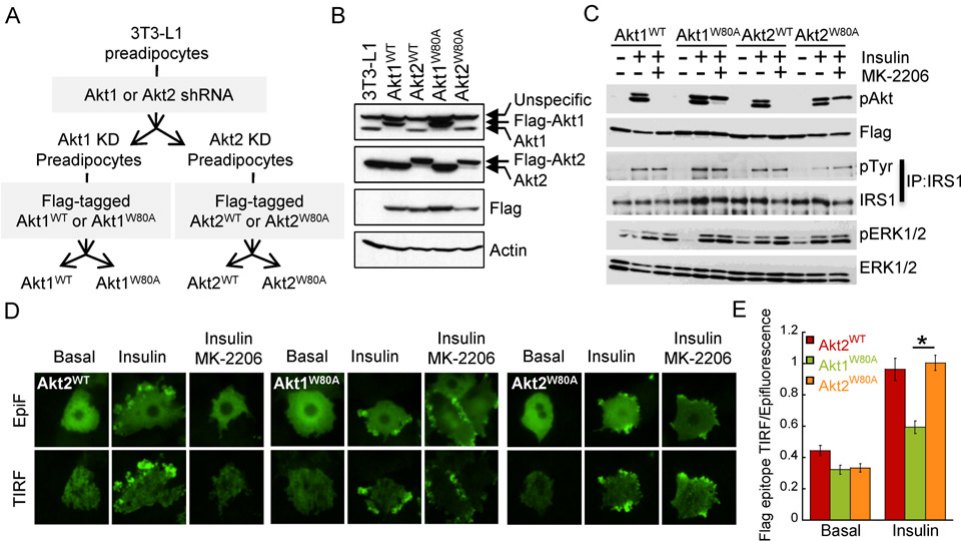
Development of adipocytes stably expressing Akt mutants resistant to MK-2206 (**A**) Schematic representation of the protocol used to develop 3T3-L1 adipocytes stably expressing Flag-tagged Akt1^WT^, Akt1^W80A^, Akt2^WT^, Akt2^W80A^. (**B**) Western blot analyses of protein extracts from adipocytes stably expressing Flag-tagged WT or W80A Akt1 and Akt2. (**C**) Immunoblot analyses of protein extracts from adipocytes expressing WT or W80A Akt1 or Akt2. Cells were treated with 1 nM insulin for 15 min. Some cells were treated with 1 μM MK-2206 for 1 h prior to insulin addition as noted. IRS1 was immunoprecipitated from cell lysates using anti-IRS1 specific antibody. (**D**) Immunofluorescence analyses of Flag-tagged Akt2^WT^, Akt1^W80A^ and Akt2^W80A^ using an anti-Flag antibody. Epifluorescence and TIRF microscopy were used to detect total and plasma membrane-targeted Akt constructs respectively. Adipocytes were starved for 2 h followed by vehicle (Basal) or 10 nM insulin stimulation for 15 min (Insulin). Some cells were pre-treated with 10 μM MK-2206 for 1 h prior to insulin addition (Insulin, MK-2206). (**E**) Quantification of insulin-induced Flag-tagged Akt2^WT^, Akt1^W80A^ and Akt2^W80A^ redistribution to the plasma membrane using TIRF microscopy. Indirect immunofluorescence of the Flag epitope in basal or 10 nM insulin-stimulated adipocytes was measured in the epifluorescence mode and in the TIRF mode. The anti-Flag TIRF is normalized to the anti-Flag fluorescence in the epifluorescence mode. Data are normalized to that of insulin-treated Akt2^W80A^ expressing cells. Each data point represents the mean ± S.E.M., *n*=28–70 cells; *P<0.01 (paired *t* test).

We hypothesized that treatment with MK-2206 in cells ectopically expressing drug resistant Akt isoforms will enable us to acutely block endogenous Akt activity and thereby restrict insulin signalling to the drug resistant, transgenic Akt isoform, providing a cellular system to interrogate isoform specific Akt signalling. To test our hypothesis we developed 3T3-L1 adipocytes, in which we replaced endogenous Akt1 and Akt2 with Flag-tagged W80A mutants of Akt1 or Akt2 or with their respective WT counterparts (Figure 1A). To avoid potential aberrant Akt activity due to overexpression of our constructs, we first depleted the endogenous Akt kinases by retroviral transduction of pre-adipocytes with shRNAs targeting Akt1 (Akt1^KD^) or Akt2 (Akt2^KD^) respectively. We next infected those cells with lentiviruses to stably express shRNA-resistant Akt1^W80A^ and Akt2^W80A^ constructs under the control of a cytomegalovirus (CMV) promoter. Akt1^KD^ pre-adipocytes were infected with lentiviral particles encoding Flag-tagged Akt1^WT^ or Akt1^W80A^; and Akt2^KD^ cells were transduced with lentivirus encoding Flag-tagged Akt2^WT^ or Akt2^W80A^ constructs. We next assessed the expression levels of endogeneous and transgenic Akt kinases in our cell lines by Western blot analyses. Protein lysates from 3T3-L1 adipocytes were used as control. As shown in Figure 1(B), endogenous Akt1 was efficiently depleted in Akt1^WT^ and Akt1^W80A^ cell lines and transgenic Flag-tagged Akt1 constructs were detected with anti-Akt1 antibody. Similarly endogenous Akt2 levels were almost undetectable in Akt2^WT^ and Akt2^W80A^ cell lysates, where Akt2 Flag-tagged constructs were detected by the anti-Akt2 specific antibody (Figure 1B). Immunodetection with the anti-Flag antibody verified the expression of Akt transgenic constructs in our cell lines.

To test the insulin responsiveness and drug resistance of our transgenic Akt kinases we stimulated our cell lines with insulin in the presence or absence of MK-2206 and performed Western blot analyses measuring Akt phosphorylation and insulin receptor downstream signalling. Insulin induced the phosphorylation of WT Akt kinases, both the transgenic constructs and the remaining endogeneous non-targeted Akt isoform, as well as the phosphorylation of Akt1^W80A^ and Akt2^W80A^ mutants. Treatment with MK-2206 inhibited insulin-induced phosphorylation of endogenous Akt and transgenic Akt1^WT^ and Akt2^WT^ (Figure 1C). In contrast, Akt1^W80A^ and Akt2^W80A^ phosphorylation was preserved in MK-2206-treated cells following insulin treatment (Figure 1C). These data indicate that in our cellular system Akt1^W80A^ and Akt2^W80A^ are dynamically regulated by insulin and resistant to the MK-2206 inhibitor. Thus, treatment of Akt1^W80A^ or Akt2^W80A^ cells with MK-2206 allows for the specific interrogation of Akt1 or Akt2 signalling respectively. Importantly, MK-2206 did not affect insulin-mediated phosphorylation of IRS1 or the extracellular-signal-regulated kinase (ERK)1/2 kinases (Figure 1C), indicating that this drug does not impair insulin signalling upstream of Akt kinases.

Akt1 inhibition by the Akti1/2 drug requires an intact PH (pleckstrin homology)-domain [24] and myristoylated Akt1 is susceptible to this inhibitor [37] suggesting that Akti1/2 might impair the association of Akt1 with the plasma membrane. To test whether MK-2206 affects the redistribution of Akt kinases to the plasma membrane in response to insulin, we performed total internal reflection fluorescence (TIRF) microscopy analyses of Flag-tagged Akt constructs in insulin-stimulated adipocytes with or without MK-2206 treatment. Insulin induced the redistribution of Akt2^WT^, Akt1^W80A^ and Akt2^W80A^ to the cell surface as shown by an increase in TIRF intensity (Figure 1D). MK-2206 treatment impaired insulin-induced cell surface recruitment of Akt2^WT^, whereas Akt1^W80A^ or Akt2^W80A^ kinases were able to accumulate at the cell surface of MK-2206 treated cells. This is consistent with the phosphorylation of those mutants by upstream kinases in the presence of MK-2206 (Figure 1D). Thus, our data indicate that MK-2206 blunts Akt activation by inhibiting stimulus-induced plasma membrane association of Akt and this effect is overcome by the W80A mutation.

In response to insulin, Akt2 accumulates at membrane compartments to a higher degree than Akt1 and this behaviour was correlated to Akt2 signalling specificity to GLUT4 trafficking [14,38,39]. To test whether Akt drug-resistant mutants retain the stimulus-induced differential subcellular distribution of WT Akt kinases, we measured the distribution of Flag-tagged Akt constructs at the plasma membrane by quantitative TIRF microscopy. In response to insulin, Akt2^W80A^ accumulated at the adipocyte cell surface as Akt2^WT^. However, insulin-induced plasma membrane accumulation of Akt1^W80A^ was significantly lower than that of Akt2^W80A^ (Figure 1E), as previously described for their WT counterparts [14]. These data indicate that drug-resistant Akt isoforms retain the differential stimulus-regulated subcellular localization characteristic of WT Akt kinases.

**Figure 2:**
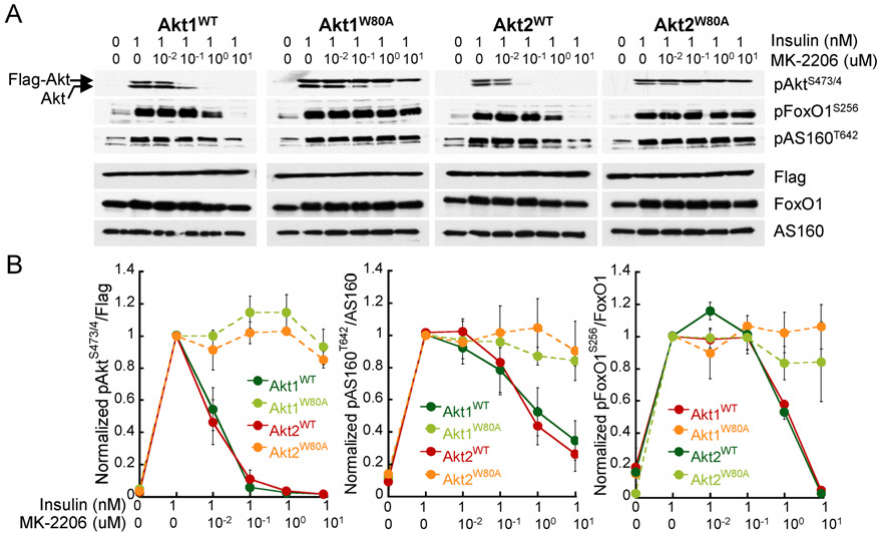
Akt1^W80A^ and Akt2^W80A^ mediate insulin signal to Akt downstream effectors (**A**) Immunoblot analyses of protein extracts from adipocytes expressing WT or W80A Akt mutants. Adipocytes were pre-treated with MK-2206 for 1 h as noted followed by stimulation with insulin for 15 min. (**B**) Densitometric analyses of immunoblots described in (**A**). For each cell line data were normalized to the insulin-treated, MK-2206 untreated condition. Each data point is the mean of 3–5 independent experiments ± S.E.M.

To assess the functionality of drug-resistant Akt mutants, we tested their ability to modulate known Akt effector molecules in response to insulin. MK-2206 treatment inhibited insulin-regulated phosphorylation of Akt1^WT^ and Akt2^WT^ and its downstream targets the Rab GAP AS160 and the transcription factor FoxO1 in a dose-dependent manner (Figures 2A and 2B). In contrast, insulin-induced phosphorylation of Akt1^W80A^ and Akt2^W80A^ remained unaffected in the presence of up to 10 μM MK-2206 and phosphorylation of AS160 and FoxO1 was not affected by MK-2206 treatment in cells expressing Akt1^W80A^ or Akt2^W80A^. Together, these data demonstrate that Akt1^W80A^ and Akt2^W80A^ mutants are resistant to MK-2206, acutely stimulated by insulin and able to signal to downstream effectors. Thus, adipocytes expressing Akt1^W80A^ and Akt2^W80A^ may provide a suitable cellular model to study insulin-regulated isoform specific Akt functions.

### Akt1 and Akt2 MK-2206 resistant mutants control insulin action in adipocytes

**Figure 3:**
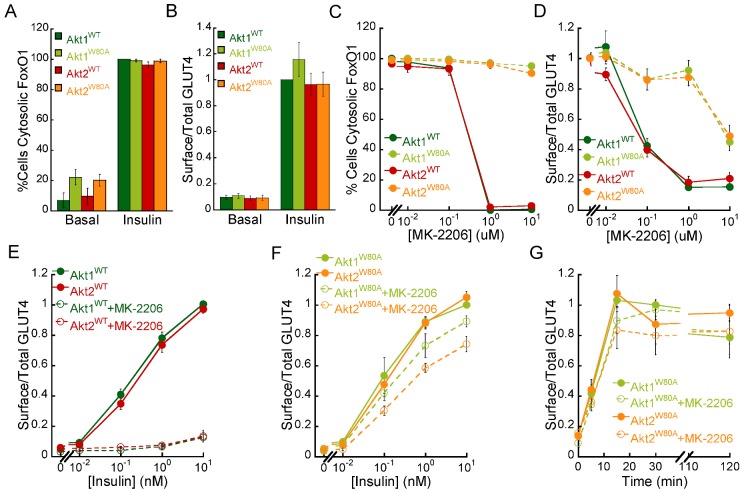
Akt1^W80A^ and Akt2^W80A^ regulate insulin-mediated FoxO1 nuclear exclusion and GLUT4 translocation (**A**) Percentage of cells expressing Akt1^WT^, Akt1^W80A^, Akt2^WT^ or Akt2^W80A^ that display cytosolic FoxO1–GFP in basal (starved) and following 1 nM insulin stimulation for 30 min. (**B**) Surface to total HA–GLUT4–GFP in basal (starved) and 1 nM insulin-stimulated adipocytes expressing Akt1^WT^, Akt1^W80A^, Akt2^WT^ and Akt2^W80A^. For each experiment surface to total GLUT4 values were normalized to that of insulin-treated Akt1^WT^ cells. Dose–response analyses of MK-2206 inhibition of insulin-mediated FoxO1–GFP nuclear exclusion (**C**) and HA–GLUT4–GFP translocation (**D**) in adipocytes. Adipocytes expressing WT or W80A Akt mutants were pre-treated with MK-2206 for 1 h as noted followed by stimulation with 1 nM insulin for 30 min. (**E**) Insulin-dose response analyses of HA–GLUT4–GFP translocation in Akt1^WT^ and Akt2^WT^ adipocytes in the presence or absence of 1 μM MK-2206. For each experiment, surface to total GLUT4 measurements were normalized to that of 10 nM insulin-treated Akt1^WT^ cells. (**F**), Insulin dose–response analyses of HA–GLUT4–GFP plasma membrane translocation in Akt1^W80A^ and Akt2^W80A^ adipocytes in the presence or absence of 1 μM MK-2206. For each experiment surface to total GLUT4 measurements were normalized to that of 10 nM insulin-treated Akt1^W80A^ cells. (**G**) Time course analyses of insulin-mediated GLUT4 translocation in adipocytes. Cells were treated with vehicle or 1 μM MK-2206 for 1 h prior to 1 nM insulin stimulation. For each experiment surface to total GLUT4 values were normalized to that of 15 min insulin-treated Akt1^W80A^ cells. All data are the average of at least three independent experiments ± S.E.M.

To validate our model system for the study of Akt isoform signalling we next investigated the role of Akt1 and Akt2 in two critical insulin-regulated processes in fat cells: the translocation of GLUT4 to the plasma membrane and the inactivation of the transcription factor FoxO1 by nuclear exclusion. Insulin regulation of GLUT4 and FoxO1 was assessed by quantitative fluorescence microscopy using HA–GLUT4–GFP and FoxO1–GFP reporters respectively. At steady-state, insulin-induced FoxO1 nuclear exclusion and GLUT4 translocation were similar in adipocytes expressing WT or MK-2206 resistant Akt mutants (Figures 3A and 3B). This indicates that in the absence of MK-2206, Akt^W80A^ mutants are functionally equivalent to their WT counterparts. We next performed MK-2206 dose–response analyses of insulin-induced FoxO1 regulation in our cell lines. MK-2206 treatment inhibited insulin-mediated FoxO1 nuclear exclusion in a dose-dependent manner in cells expressing Akt1^WT^ or Akt2^WT^. However, insulin regulation of FoxO1 was preserved in Akt1^W80A^ and Akt2^W80A^ cells (Figure 3C). These results demonstrate that both Akt1 and Akt2 signalling modulate FoxO1 nuclear exclusion in response to insulin, as previously described [34].

To assess the role of Akt isoforms in insulin-regulated GLUT4 trafficking we performed MK-2206 dose–response analyses of insulin-mediated GLUT4 translocation in our cell lines. MK-2206 treatment of adipocytes expressing Akt1^WT^ or Akt2^WT^ inhibited insulin-induced GLUT4 translocation to the plasma membrane in a dose-dependent manner (Figure 3D). Surprisingly, insulin-induced subcellular redistribution of GLUT4 was preserved in MK-2206 treated Akt1^W80A^ and Akt2^W80A^ adipocytes, indicating that both Akt kinases mediate insulin signalling to GLUT4. High MK-2206 doses (>10^3^ IC50 for Akt1 and Akt2 inhibition) partially inhibited insulin-induced GLUT4 translocation in Akt1^W80A^ and Akt2^W80A^ cells, despite the fact that under those conditions the phosphorylation of Akt mutant kinases was largely preserved (Figure 2), suggesting potential Akt-independent effects of the inhibitor at those high doses. Nonetheless, our results indicate that both Akt1^W80A^ and Akt2^W80A^ are able to acutely regulate GLUT4 trafficking, in contrast with the Akt2-specific requirement for GLUT4 regulation previously described using Akt isoform specific knockout or knockdown cells [11–14].

To further characterize the contribution of Akt isoform specific signalling to insulin regulated GLUT4 trafficking we performed insulin dose–response and time course analyses of GLUT4 plasma membrane translocation in combination with MK-2206 treatments. Insulin-induced GLUT4 translocation was inhibited at every insulin dose by 1 μM MK-2206 treatment in cells expressing Akt1^WT^ and Akt2^WT^ (Figure 3E). On the other hand, insulin dose–response for GLUT4 translocation was largely preserved in cells expressing either Akt1^W80A^ (EC50_control_=0.100 nM; EC50_MK-2206 treated_=0.152 nM) or Akt2^W80A^ (EC50_control_=0.155 nM; EC50_MK-2206 treated_=0.256 nM; Figure 3F), indicating that both kinases are equally able to acutely regulate GLUT4 trafficking. Time course analyses of insulin-mediated GLUT4 translocation in Akt1^W80A^ and Akt2^W80A^ expressing cells showed that Akt1 and Akt2 drug-resistant mutants promote and sustain GLUT4 translocation to the plasma membrane for up to 2 h indistinguishably (Figure 3G). Thus, together our results demonstrate that both Akt1^W80A^ and Akt2^W80A^ control GLUT4 trafficking in response to insulin, providing evidence for a redundant role of Akt kinases in the acute regulation of glucose transport.

### Akt1 signalling modulates adipocyte differentiation

**Figure 4:**
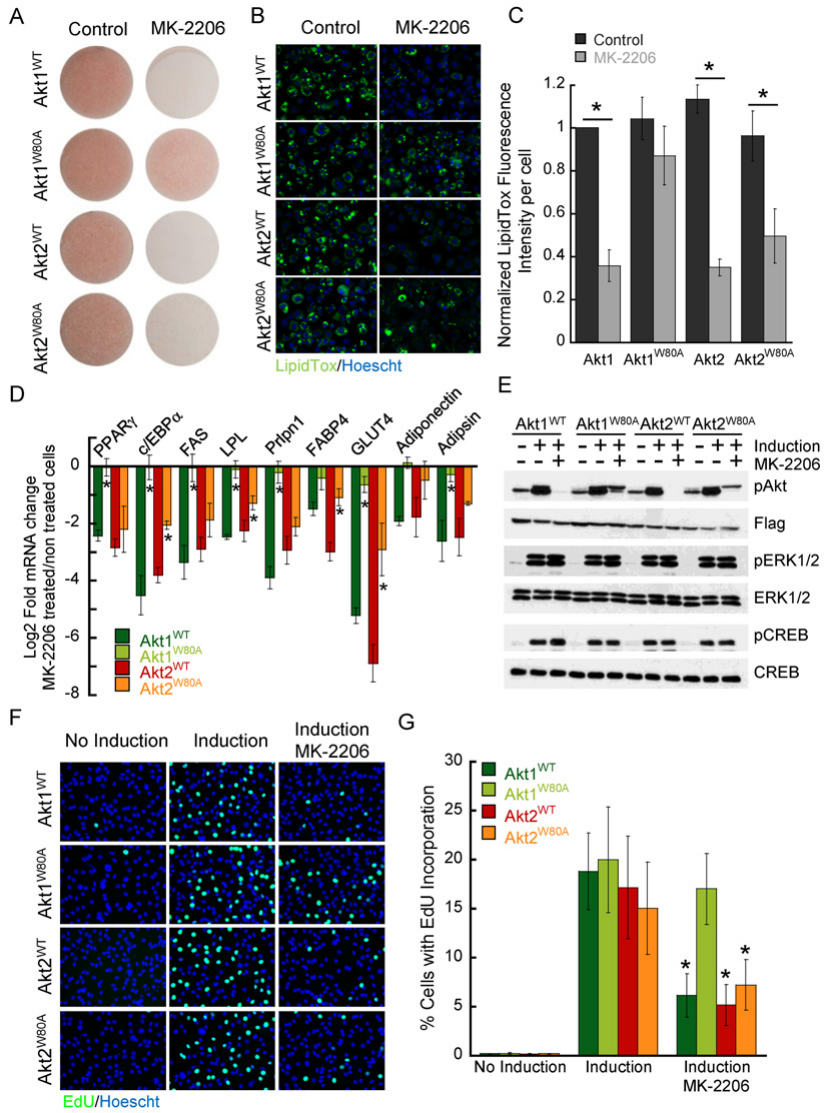
Akt1-specific signalling controls adipocyte differentiation (**A**) Oil–Red-O staining of adipocytes expressing WT or W80A Akt mutants differentiated in the presence or absence of MK-2206. Neutral lipid staining (**B**) and quantification (**C**) using LipidTox green stain in adipocytes expressing WT or W80A Akt mutants differentiated in the presence or absence of MK-2206. Bars are the mean of four independent experiments ± S.E.M. *P<0.01 (paired *t* test) (**D**) RT-qPCR analyses of adipocyte markers in cells expressing WT or W80A Akt constructs differentiated into adipocytes in the presence or absence of MK-2206. Gene expression was normalized to the housekeeping gene *RPLP0*. Data represent the log_2_-fold change in gene expression in MK-2206-treated compared with -untreated conditions. Data are the mean of 3–6 independent experiments ± S.E.M. *P<0.05 (ANOVA). Statistical significance for the data comparisons Akt1^W80A^ compared with Akt1^WT^ and Akt2^W80A^ compared with Akt2^WT^ is shown. (**E**) Immunoblot analyses of protein extracts derived from pre-adipocytes expressing WT and W80A Akt isoforms. Confluent pre-adipocytes were starved for 2 days and then treated with induction medium for 15 min with or without 2 μM MK-2206. (**F**) EdU incorporation in pre-adipocytes expressing WT or W80A Akt1 and Akt2 constructs. Cells were kept for 16 h in the presence of growth media (no induction), differentiation media (induction) or differentiation media plus MK-2206 (induction, MK-2206), followed by a 2-h incubation with EdU. Nuclei were counterstained with Hoechst. (**G**) Percentage of cells with EdU incorporation determined by fluorescence microscopy. Data are the mean of four independent experiments ± S.E.M. *P<0.01 (paired *t* test induction compared with induction/MK-2206).

Previous *in vitro* studies proposed a specific role for Akt1 signalling in the regulation of adipogenesis [40,41]. To further validate the applicability of our model system to study isoform specific Akt signalling, we next tested the role of Akt1 and Akt2 in adipocyte differentiation. Pre-adipocytes expressing WT or W80A mutant Akt1 and Akt2 were induced to differentiate in the presence or absence of MK-2206 and we then measured lipid accumulation, a hallmark of adipocyte differentiation, by Oil-Red-O and LipidTox green staining. Treatment with MK-2206 during adipogenic induction inhibited triglyceride accumulation in cells expressing Akt1^WT^ or Akt2^WT^ (Figures 4A–4C). Differentiation-induced lipid accumulation in Akt1^W80A^ cells was not affected by MK-2206 treatment, whereas it was significantly decreased in Akt2^W80A^ cells (50.9±14.3%; Figure 4C), indicating that Akt1 signalling is specifically required for adipogenesis.

To corroborate those results we measured transcription markers of the adipogenic differentiation programme by RT-qPCR analyses in cells differentiated in the presence or absence of MK-2206. Inhibition of Akt signalling during adipocyte differentiation blunted the up-regulation of a broad range of adipocyte markers in Akt1^WT^ or Akt2^WT^ cells (Figure 4D; Supplementary Figure S2). Adipogenic gene induction was not affected by MK-2206 in Akt1^W80A^ cells, whereas it was impaired in Akt2^W80A^ cells, although MK-2206 inhibition of some adipose specific genes (i.e. c/EBPα and GLUT4) was partially ameliorated in Akt2^W80A^ cells compared with Akt2^WT^ cells (Figure 4D). These results further support the conclusion that Akt1 signalling is critically required for adipogenesis, whereas Akt2 signalling might only partially contribute to full adipocyte specification.

To interrogate whether MK-2206 effects on adipogenesis were specific to Akt signalling inhibition we performed Western blot analyses of pre-adipocyte protein lysates upon differentiation induction in the presence or absence of MK-2206. As shown in Figure 4(E), MK-2206 treatment inhibits Akt phosphorylation without affecting the phosphorylation of the MAP kinases ERK1/2 and the transcription factor CREB (cAMP response element-binding protein), two critical signalling intermediates required for adipogenesis.

Prior to committing to terminal differentiation, pre-adipocytes undergo a mitotic clonal expansion (MCE), thought to be controlled by Akt1 signalling [40]. To further validate our model system, we tested the contribution of Akt isoforms to the MCE of pre-adipocytes. We induced pre-adipocytes to differentiate in the presence or absence of MK-2206 for 16 h, followed by an EdU pulse. We then measured EdU incorporation by fluorescence microscopy. Treatment with induction medium promoted cell cycle re-entry in pre-adipocytes as indicated by increased EdU incorporation and this process was blunted in MK-2206 treated Akt1^WT^ (70±12%), Akt2^WT^ (73±11%) and Akt2^W80A^ (54±13%) cells (Figures 4F and 4G). On the other hand, MK-2206 did not significantly affect EdU incorporation in Akt1^W80A^ pre-adipocytes in response to induction medium (Figures 4F and 4G). These results demonstrate a specific requirement for Akt1 signalling during the MCE that precedes pre-adipocyte commitment to terminally differentiate.

To further investigate the specificity of Akt1 signalling in the control of adipogenesis we tested whether increasing levels of expression of Akt2^W80A^ could overcome Akt1 signalling specific requirement during the MCE of pre-adipocytes. To obtain cell populations with different levels of Akt2^W80A^ expression we sorted our Akt2^W80A^ expressing cells by flow cytometry into three different populations containing low (Akt2^W80A^_Low_), medium (Akt2^W80A^_Medium_) and high (Akt2^W80A^_High_), expression levels of the tdTomato marker. Western blot analyses of protein lysates from those cell populations revealed that expression levels of Flag–Akt2^W80A^ in Akt2^W80A^_High_ cells was increased by ~2-fold compared with Akt2^W80A^_Medium_, Akt2^W80A^_Low_ and our initial Akt2^W80A^ cell populations, as well as compared with endogenous Akt2 levels in 3T3-L1 pre-adipocytes (Supplementary Figure S3A). We next performed EdU incorporation measurements in pre-adipocytes from those cell populations induced to differentiate in the presence or absence of MK-2206. Differentiation medium induced EdU incorporation was largely preserved in MK-2206 treated Akt1^W80A^ cells. On the other hand, MK-2206 treatment blunted EdU incorporation in all Akt2^W80A^ cell populations, irrespective of the expression levels of the Akt2^W80A^ transgene (Supplementary Figure S3B), further supporting a requirement for Akt1 signalling in regulating the MCE that precedes adipocyte differentiation. Together, these data further indicate that Akt1^W80A^ and Akt2^W80A^ mutants preserved isoform signalling specificity of their WT counterparts and validate our model system as a new approach to delineate the molecular mechanisms underlying adipocyte differentiation.

## DISCUSSION

Akt kinases are critical signal transducers that govern glucose and lipid homoeostasis in response to insulin [2,3]. Consistent with the pleiotropic effects of Akt kinases in metabolic control, deregulation of Akt signalling is a hallmark of insulin-resistant tissues [3,42]. Still, the molecular mechanisms by which Akt isoforms control lipid and glucose metabolism are not well understood. Genetic studies revealed a requirement for Akt2-specific signalling in the maintenance of glucose homoeostasis [8–10]. However, due to different expression levels of individual Akt isoforms in metabolic tissues, compensatory mechanisms and/or functional redundancies the molecular mechanisms and effectors that dictate Akt isoform signalling specificity in insulin action remain to be identified. To develop such knowledge, we would require model systems that allow us to acutely inhibit the function of individual Akt isoforms and to biochemically interrogate their metabolic functions.

In the present study, we employed a chemical genetic approach to develop a novel model system that allows for acute control of isoform-specific Akt signalling. Previously, small molecules that allosterically inhibit Akt1 and Akt2 function have been discovered [23,28]; and it was shown that conversion of Trp^80^ to alanine in Akt1 confers resistance to one of these inhibitors, the quinoxaline Akti1/2 [24]. Our data revealed that W80A conversion also renders both Akt1 and Akt2 insensitive to the MK-2206 inhibitor, a newer drug with enhanced affinity and improved target specificity [27,28]. Consistent with our data, recent molecular docking studies of Akt1 and MK-2206 identified W80 as a residue that mediates their interaction [43]. Importantly, whereas Akt1^W80A^ and Akt2^W80A^ mutants are both resistant to MK-2206, they are still dynamically regulated by insulin and their phosphorylation of downstream effectors is indistinguishable from that of their WT counterparts. These features make Akt1^W80A^ or Akt2^W80A^ mutants useful tools for the study of isoform-specific Akt signalling when they replace the endogenous counterpart in a cellular system. Our data show that treatment of adipocytes expressing drug-resistant Akt mutants with MK-2206 inhibits the remaining endogenous Akt kinase whereas the corresponding Akt1^W80A^ or Akt2^W80A^ remain active, thereby restricting Akt's signalling responses to one specific isoform.

Our new model system provides important advantages for the investigation of isoform-specific Akt functions since it: (a) provides acute control of Akt1 and Akt2 function and therefore avoids artifacts due to compensatory mechanisms associated with long-term deletion of Akt kinases; (b) avoids off-target effects inherent to pharmacological interventions due to the isoform specific rescue by the drug resistant kinase; (c) allows for controlled expression levels of transgenic Akt kinases to test the contribution of expression levels compared with signalling specificity to Akt isoform-specific functions and (d) provides a cellular system suitable for molecular, biochemical and MS studies to identify Akt isoform functions and effectors. Thus our experimental approach provides a robust cellular system to study Akt isoform-specific metabolic functions and to pursue the biochemical identification of the molecular effectors responsible for isoform-specific Akt signalling.

Using our model system, we describe an essential role for Akt1 signalling in adipocyte differentiation, consistent with previous adipogenesis studies in Akt1- and Akt2-deficient mouse embryonic fibroblasts [40,41]. Our data reveal that acute inhibition of Akt1 signalling during adipocyte differentiation blunts activation of the transcriptional programme that defines the characteristics of fat cells and enables lipid accumulation. Specifically, our data suggest that Akt1 signalling is essential for the MCE of pre-adipocytes prior to their commitment to terminal differentiation. To this end, our data support a previous report by Zhang and colleagues [37], which proposed that Akt1 signalling promotes cell cycle progression of arrested pre-adipocytes by inhibiting FoxO1 and restricting the expression of its target genes including the cell cycle inhibitor p27^kip1^ [40]. However, our data also suggest a potential contribution of Akt2 signalling in regulating the adipogenic transcriptional programme, because Akt2^W80A^ was able to partially rescue the expression of some adipocyte markers such as c/EBPα and GLUT4, even though a fully differentiated state was not achieved. This observation is consistent with previous reports which suggested that genetic deletion of both Akt1 and Akt2 is required to completely block the formation of adipose tissue in mice [44]. Additional studies investigating short- and long-term effects of Akt kinases on adipogenesis in response to receptor-mediated signalling will be required to strengthen our molecular understanding of the processes involved in fat cell specification. Our development of a model system that enables the temporal control of Akt isoform signalling now offers a robust experimental platform to interrogate the molecular mechanisms by which signalling by specific Akt isoforms govern adipogenesis.

Analyses of insulin action in our new adipocyte model system confirmed that Akt1 and Akt2 can both regulate FoxO1 in response to insulin as previously proposed [34]. Likewise, our acute inhibition studies showed that both, Akt1 and Akt2 are also able to regulate GLUT4 trafficking. This result however is different from previous reports, including our own, which suggested a specific role for Akt2 in GLUT4 regulation when Akt1 or Akt2 function has been inhibited by gene knockout or knockdown [9,11–14,31]. How can we explain this discrepancy between previous and current observations? One possibility is that mutation of Trp^80^ alters the specificity of Akt isoforms. However, our data argue against that hypothesis because in response to insulin, Akt2 accumulates at membrane compartments to a higher degree than Akt1, a behaviour that has been correlated with Akt2's ability to regulate GLUT4 trafficking [14,38,39]. We also found that Akt2^W80A^ is retained at the cell surface to a higher extent than Akt1^W80A^ in insulin-treated cells. Thus, Akt1^W80A^ and Akt2^W80A^ display specific characteristics of their WT counterparts, yet both kinases are able to control GLUT4 trafficking in fat cells. In addition, Akt1^W80A^, but not Akt2^W80A^, was able to promote adipogenesis, which further indicates that Akt1^W80A^ and Akt2^W80A^ mutants retain the signalling specificity of WT Akt isoforms. Another simple explanation for the discrepancy between the data is that Akt2 expression levels are higher than Akt1 expression levels in fat, muscle and liver cells. In our system transgenic Akt kinases are expressed at similar levels, allowing us to directly compare the ability of Akt isoforms to transduce insulin signalling. Yet, ectopic expression of Akt1 is not sufficient to rescue the defect in GLUT4 translocation of Akt2-null adipocytes suggesting that differential Akt isoform expression levels might not solely determine Akt2's preferential control of glucose transport *in vivo* [13,14]. It is also possible that Akt2 could have scaffolding functions, apart from its kinase activity, which are required to mediate GLUT4 translocation. Those functions could remain preserved in our system where endogenous Akt2 is present, but inhibited by the drug. Alternatively, long-term deletion of Akt2 may not only impair acute insulin signalling to GLUT4 trafficking, but also alter the transcriptional regulation or protein stability of components of the insulin signalling pathway or the GLUT4 trafficking machinery, independent of their acute functions in response to insulin. Based on our observation that both Akt1 and Akt2 can acutely mediate insulin signalling to GLUT4 trafficking, further studies will be required to identify the mechanisms by which Akt2 may preferentially regulate glucose transport. The temporal and reversible control of isoform-specific Akt signalling, as offered by our system, might provide a valuable tool to mechanistically interrogate Akt isoform-specific control of glucose uptake at the biochemical level.

In summary, we have developed and validated a novel cellular model system that allows for specific and acute regulation of Akt1 and Akt2 signalling. Using this system, we show that Akt1 and Akt2 are able to acutely control insulin signalling to GLUT4 trafficking, whereas Akt1 signalling is specifically required for the early stages of adipogenesis. Combination of our model system with quantitative phospho-proteomic and global transcriptomic analyses would provide a robust experimental platform for the future identification of isoform-specific Akt effector molecules, a critical step to enhance our understanding of insulin's metabolic functions and for the development of advanced pharmacological strategies to improve insulin action.

## Abbreviations

AS160: Akt substrate of 160 kDa
DMEM: Dulbecco's modified Eagle's medium
ERK: extracellular-signal-regulated kinase
FoxO1: forkhead box O1
GLUT: glucose transporter
HA: haemagglutinin
IRS1: insulin receptor substrate 1
MCE: mitotic clonal expansion
PI3-kinase: phosphatidylinositol 3-kinase
PKB: protein kinase B
MAPK: mitogen-activated protein kinase
mTOR: mammalian target of rapamycin
RT-qPCR: real-time quantitative PCR
TIRF: total internal reflection fluorescence
WT: wild-type
